# Binding modes of 2,4-diaminoquinazoline and 2,4-diaminopteridine analogs to *P. falciparum* dihydrofolate reductase enzyme: Molecular docking studies

**DOI:** 10.4103/0250-474X.70478

**Published:** 2010

**Authors:** L. Adane, P. V. Bharatam

**Affiliations:** Department of Medicinal Chemistry, National Institute of Pharmaceutical Education and Research (NIPER), S.A.S. Nagar-160 062, India

**Keywords:** Dihydrofolate reductase, molecular docking, multiple mutations, Pyrimethamine-resistant *P. falciparum*, 2,4-diaminoquinazoline

## Abstract

A molecular docking study was carried out on 28 compounds belonging to 2,4-diaminoquinazoline and 2,4-diaminopteridine analogs using Glide, FlexX and GOLD programs and the X-ray crystallographic structures of the quadruple mutant (1J3K:pdb) and wild type (1J3I:pdb) *Plasmodium falciparum* dihydrofolate reductase enzyme. The experimental conformation the bound ligand WR99210 was precisely reproduced by the docking procedures as demonstrated by low (<2.00 Å) root-mean-square deviations. The results indicated that most of the compounds dock into the active sites of both the wild type and quadruple mutant *P. falciparum* dihydrofolate reductase enzymes. Visual inspection of the binding modes also demonstrated that most of the compounds could form H-bond interactions with the key amino acid residues (Asp54, Ile14 and Leu/Ile164) and with better docking scores than the bound compound (5). Their long side chains orient in the hydrophobic portion of the active site which is occupied by trichloro aryloxy side chain of WR99210 (5). Thus, avoid potential steric clashes with Asn108 (mutated from Ser108). Such a clash is known to be responsible for the resistance of the *P. falciparum* to pyrimethamine and cycloguanil.

Malaria is one of the most prevalent parasitic diseases in tropical regions of the world which causes about 300-500 million clinical cases and 1.5-3 million deaths per year[1–5]. Emergence of drug-resistant *P. falciparum* parasite is one of the major factors responsible for today’s widespread occurrence of malaria which compromised clinical uses of the available antimalarial drugs such as chloroquine (1), cycloguanil (2) and pyrimethamine (3)[6–13]. The chemical structures of chloroquine and some antifolate-based antimalarial agents are given in fig. 1. In order to address the problem of drug resistance, a number of strategies have been employed which include combination therapy, identification and validation of new targets in the parasitic cells, and designing new compounds/drugs for malaria chemotherapy. However, the successes are not as such promising as they are expected to be. Thus, there is an urgent need of new antimalarial agents which could effectively inhibit drug resistant *P. falciparum* parasite.

**Fig. 1:**
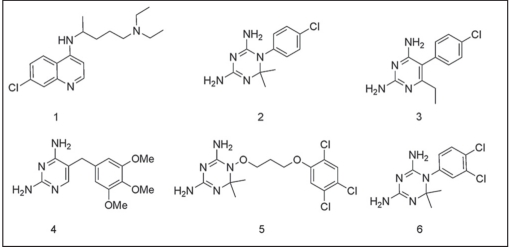
Chemical structures of chloroquine (1) and main type-2 antifolates (2-6)

Dihydrofolate reductase (DHFR) domain of the *P. falciparum* bifunctional enzyme known as dihydrofolate reductase-thymidylate synthase (DHFR-TS) is one of the validated targets in malaria chemotherapy[14–18]. This enzyme catalyzes the nicotine amide adenine nucleotide phosphate (NADPH) dependent reduction of dihydrofolate (DHF) to tetrahydrofolate (THF) which is essential for DNA synthesis. Inhibition of DHFR enzyme effectively interrupts DNA synthesis which ultimately leads to the parasitic cell death. Thus, this enzyme is a specific target of antifolate-based antimalarial agents such as 2 and 3. However, due to rapid emergence and spread of drug-resistant strains of the parasite, the therapeutic values of these drugs have dramatically reduced in many parts of the world particularly in sub-Saharan Africa, Latin America, southern Asia and Oceania.

Homology modelling[19–23] and X-ray crystallographic[24] studies clearly indicated that accumulation of genetic mutations at one or more amino acid residues 16, 51, 59, 108 and 164 are responsible for antifolate resistance. *P. falciparum* parasite harboring mutations Ala16Val+Ser108Thr showed resistance to 2 but sensitive to 3 whereas a single mutation S108N causes resistance to 3 which is also enhanced by additional mutations, Asn51Ile+Cys59Arg. On the other hand, the *Pf*DHFR enzyme carrying mutations at amino acid residues 51, 59, 108 and 164 shows cross-resistance to both drugs (i.e., 2 and 3)[19–26]. In all the cases, mutation causes steric clashes between the bulky (mutated) amino acid residues and antifolate drugs. Such steric clashes displace the drugs from their optimal orientation. The displacement, in turn, reduces the strength of the inhibitors’ interactions with the residue Asp54 which is crucial for inhibitor binding. This consequently results in the loss of inhibitory activities of antifolate drugs. For instance, in the parasite harboring Ala16Val+Ser108Thr mutated DHFR enzyme, one of the methyl groups at the 6-position of 2 experiences a severe steric interaction from V16. Such an interaction decreases the binding affinity of 2. In the case of *Pf*DHFR enzyme with multiple mutations, steric clashes of the para-Cl groups of both 2 and 3 with Asn108 (mutated from Ser108) result in the loss of the inhibitory activities of these drugs[20]. It has also been observed that multiple mutations cause synergism to the parasite’s resistance. Several structure-activity relationship studies[27–32] and reviews[33,34] have been published that helped in a better understanding of molecular level mechanisms of *P. falciparum* parasite resistance to antifolates.

Molecular docking methods are widely used by pharmaceutical industries and academic institutes to study drug-target interactions in order to understand the basic electronic/steric features required for therapeutic action and to design new drug candidates with improved activities. The information generated from docking calculations help to get insight into interactions of ligands with amino acid residues in the binding pockets of targets, and also used to predict the corresponding binding affinities of ligands[35]. As structure-based drug design approach, these methods are being employed in the discovery of *Pf*DHFR enzyme inhibitors which possess the required chemical properties for steric and electrostatic complementarity between the ligand and the site of action of the target enzyme. Toyoda *et al*. used molecular docking studies to identify potential antimalarial agents such as 2-amino-1,4-dihydro-4,4,7 8-tetramethyl-s-triazino[1,2-a] benzimidazole and pyridoindole from commercially available compounds[36]. Rastelli *et al*. also used this approach to discover new classes of *Pf*DHFR enzyme inhibitors which are structurally different from the classical antifolates[37]. Dasgupta *et al*. carried out a high-throughput *in silico* screening of database with consequent *in vitro* enzymatic assay and cellular culture studies[38]. They identified three novel biguanide analogs which were found to be active against both the wild type and quadruple mutant *Pf*DHFR enzymes. Fogel *et al*. also employed the same approach to study the binding interactions of analogs of 3 in the active sites of the wild type and multiple drug-resistant *Pf*DHFR enzymes[39]. Molecular docking also helps in target guided design and synthesis of lead compounds which could be developed as inhibitors of DHFR enzymes from different species including wild type and mutant strains of *Pf*DHFR enzymes[40].

Recently Ommeh *et al*. reported the antiplasmodial activities of the title compounds against the multiple drug resistant (V1/S) strain of *P. falciparum*[41]. Based on their observations of the *in vitro* activity test of 7 in combination with dapsone, Ommeh *et al*. suggested that these compounds could competitively bind in the active site of the DHFR enzyme of *P. falciparum*, and act as DHFR inhibitors. But no molecular docking studies were carried out by them or other research groups to substantiate this suggestion. Thus, we were interested to study the binding modes of these compounds in the binding regions of quadruple mutant and wild type *Pf*DHFR enzymes in order to get insight into their binding modes and affinities. The chemical structures of the compounds used in the study have 2,4-diamino functional groups, and are expected to form hydrogen bond interactions with amino acid residues in the active site of the *Pf*DHFR enzyme (figs. 2 and 3). Most of the compounds also have potentially flexible side chains. To the best of our knowledge, no similar study has been reported. This prompted us to carry out the present study in order to examine their binding interactions and orientations in the active sites of the above mentioned enzymes. Thus, the binding modes (orientations), scores and their interactions with key amino acid residues will be used for the discussion.

**Fig. 2:**
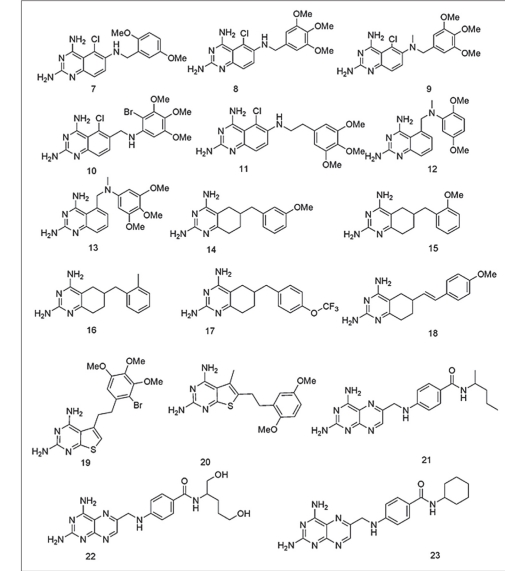
The chemical structures of the compounds used in the study

**Fig. 3:**
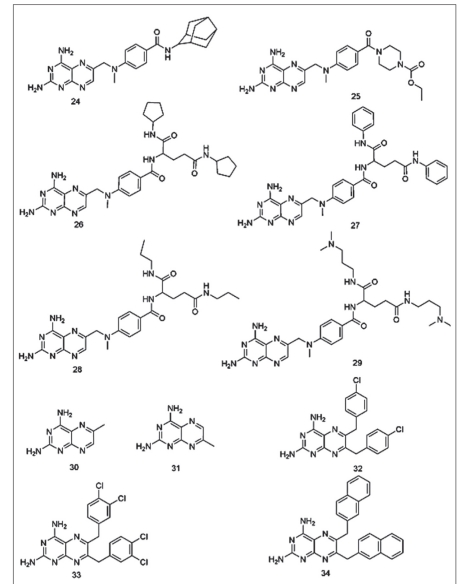
The chemical structures of the compounds used in the study

In this paper, we present a comparative analysis of binding modes and interactions of these compounds with the corresponding scores, orientation and interactions of WR99210 (5) in the X-ray crystal structure of the quadruple mutant *Pf*DHFR-inhibitor complex[24]. FlexX[42,43], GOLD[44] and Glide[45,46] docking programs were used to calculate the binding scores and modes of the compounds (see experimental section). Structure-activity relations (SAR) analyses are also used to correlate the reported inhibitory activities and the observed docking results.

## MATERIALS AND METHODS

### Data set of compounds:

A data set of 28 compounds consisting of 2,4-diaminoquinazoline, 2,4-diamino-5,6,7,8-teterahydroquinazoline and 2,4-diaminopteridine analogs were used in this study. The *in vitro* growth inhibitory activities of the compounds against multiple drug-resistant (V1/S strain) *Pf*DHFR enzyme have been reported in literature[41]. The reported IC_50_ values of the compounds were converted in to pIC_50_ using the formula -logIC_50_ for the purpose of discussion.

### Ligand structure preparation:

The compounds used in the study were built using the SKETCH module of SYBYL7.1 and subjected to 1000 cycle minimization with standard Tripos force field and 0.005 kcal/mol.Å energy gradient convergence criterion using Powell’s method[47]. The molecules were then saved as. mol2 files and used as input for the FlexX and GOLD docking calculations. For Glide docking calculations, the ligand preparation was performed using the Schrodinger Suite 2007, including Maestro 8.0, Glide 4.5[48].

### Protein structure preparation:

The X-ray crystallographic structures of the wild type (1J3I:pdb)[24] and quadruple mutant *Pf*DHFR (1J3K:pdb)[24] enzymes were obtained from the RCSB protein databank. They were used in order to model the protein structure in this study. Both structures contained compound 5 bound to the active site in the presence of NADPH. During preparation of the receptor description file (.rdf) for FlexX docking, a radius of 6.5 Å around the bound ligand was used to define the active site[47]. On the other hand, for GOLD docking studies, DHFR structures were truncated to a 10 Å radius from each atom in the bound inhibitor, 5, to simplify the calculations. Then hydrogen atoms were added and all water molecules were removed. The resulting proteins were saved as. mol2 file to be used as input for the docking calculations[49]. For the Glide docking studies, only chain A and cofactor NDP610 of *Pf*DHFR-TS were retained. All water molecules and the rest of the chains (B, C and D) were removed from the complex, and the protein was minimized using the protein preparation wizard. Partial atomic charges were assigned according to the OPLS-AA force field. A radius of 15 Å was selected for active site cavity during receptor grid generation[48]. It is important to know that the way of defining active site in the above three softwares is completely different. This is the reason why three different radius values are given in defining the size of active site of the *Pf*DHFR enzyme used in the study. The reported radius values are optimized values to reproduce the bound ligand (5) conformation.

### Docking protocols and their validation:

For the FlexX docking calculation, default parameters were used and 30 solutions were generated for each compound. In order to verify reproducibility of the docking calculations, the bound ligand (5) was extracted from the complexes and submitted for one-ligand run calculation. This reproduced top scoring conformations of 5 falling within rmsd values of 1.13 and 0.96 Å from the bound X-ray conformation for wild type and quadruple mutant *Pf*DHFR enzymes, respectively. For the GOLD docking studies, all the studies were performed using the default GOLD fitness function (VDW= 4.0, H-bonding= 2.5) and default evolutionary parameters: population size= 100; selection pressure= 1.1; number of operations= 100,000; number of islands= 5; niche size= 2; migration= 10; mutation= 95; crossover= 95[44]. One of the carbonyl oxygen atoms on Leu164 and Ile14 in quadruple mutant and wild type *Pf*DHFR, respectively, were selected as the binding site centers for all calculations. Before doing actual docking calculations of the study compounds, the reproducibility of docking protocol was evaluated by docking 5 into the prepared active sites of both enzymes. The resulting top scoring conformations of 5 were found to fall within root-mean-square deviations (rmsd) values of 1.312 Å and 0.871 Å from the bound X-ray conformation in wild type and quadruple mutant X-ray crystal structures, respectively. Fifty docking runs were performed per structure unless 3 of the 50 poses were within 1.5 Å rmsd of each other. The outputs were exported to Silver window for visual inspection of the binding modes and interactions of the compounds with amino acid residues in the active sites. For the Glide docking, Glide v4.5 was used for the calculations. Similar to the other two docking approaches, the reproducibility of the method was evaluated by docking the bound ligand into the prepared active sites. The top scoring conformations of the bound ligand were found to be 0.374 and 0.489 Å for wild type and quadruple mutant *Pf*DHFR, respectively, suggesting that the method is valid enough to be used for docking studies of other compounds. Output was set to give 10 docking poses/ligand whereas default values were used for other parameters[45,46]. Flexible docking option and XP mode were used in all the calculations.

## RESULTS AND DISCUSSIONS

A data set consisting of four classes of compounds, namely, 2,4-diaminoquinazoline, 2,4-diamino-5,6,7,8-teterahydroquinazoline, 2,4-diaminothieno2,3-d] pyrimidines and 2,4-diaminopteridine were used for these docking studies (figs. 2 and 3). All the compounds were subjected to FlexX, GOLD and Glide docking calculations using the active sites of wild type and quadruple mutant *Pf*DHFR enzymes. Table 1 shows the binding scores of the compounds in the active site of quadruple mutant *Pf*DHFR enzyme.

**TABLE 1:** DOCKING SCORES OF THE STUDY COMPOUNDS IN THE ACTIVE SITE OF QUADRUPLE MUTANT *Pf*DHFR.

Compound	Glide score	FlexX score	GOLD fitness score	pIC_50_, nM^f^
5 (ref)	-8.44*a* (-80.20)*	-17.86*a* (4)**	70.21*a*	8.57
7	-11.43*a* (-80.1)	-25.56*c* (3)	64.24*c*	8.05
8	-11.51*a* (-91.3)	-22.51*c* (3)	64.66*c*	7.49
9	-11.97*a* (-84.9)	-24.76*a* (3)	65.31*c*	7.51
10	-11.70*a* (-89.1)	-25.43*b* (4)	68.92*c*	6.65
11	-11.93*a* (-72.6)	-22.08*a* (2)	64.77*a*	5.85
12	*dd*	*dd*	*dd*	5.77
13	*dd*	*dd*	61.55*a*	5.00
14	-11.02*a* (-64.9)	-23.26*a* (4)	55.69*a*	7.54
15	-10.45*a* (-66.4)	-27.44*a* (5)	55.25*a*	7.44
16	-10.26*a* (-65.1)	-26.48*a* (5)	57.47*a*	7.30
17	-11.06*a* (-66.70)	-25.01*a* (5)	66.58*a*	6.85
18	*dd*	-22.06*a* (5)	*dd*	6.77
19	-10.966*a* (-85.0)	-16.00*c* (5)	59.30*a*	5.00
20	-12.22*a* (-74.70)	-25.49*a* (4)	59.45*a*	5.00
21	-12.06*a* (-86.70)	-39.79*a* (3)	67.95*b*	5.77
22	-13.52*a* (-103.0)	-41.24*b* (5)	62.86*a*	5.55
23	-12.14*a* (-93.10)	-40.02*b* (3)	70.34*a*	5.89
24	-10.21*a* (-93.10)	-37.29*a* (3)	64.20*d*	5.75
25	-11.93*a* (-90.6)	-32.24*a* (4)	64.81*a*	6.00
26	-12.76*a* (-128.70)	-45.43*a* (4)	*dd*	6.20
27	-10.37*a* (-139.0)	-53.66*a* (3)	*dd*	5.92
28	-12.25*a* (-127.6)	-46.87*a* (2)	*dd*	5.85
29	-6.87*a* (-103.1)	-46.55*a* (2)	*dd*	5.00
30	-7.80*a* (-49.7)	-18.63*a* (5)	34.42*a*	5.00
31	-7.96*a* (-46.10)	-22.06*a* (5)	38.97*a*	5.00
32	*dd*	*dd*	*dd*	5.00
33	*dd*	*dd*	*dd*	5.00
34	*dd*	*dd*	*dd*	5.00

a Compounds showing H-bond interactions with Asp54, Ile14, Leu164;

b compounds showing H-bond interactions with Asp54, Ile14, Leu164 and Asn108;

c compounds showing H-bond interactions with Asp54 and Ile14 or Leu164;

d compounds showing H-bond interaction with only Asp54;

e compounds showing H-bond interaction with Asp54 and Asn108.

dd compounds didn’t dock into the active site;

f IC_50_ values are reported in ref. 37;

* numbers in the parentheses indicate Emodel values;

** numbers in the parentheses indicate Cscore values.

Visual inspection was performed on the resulting docking solutions of the compounds in order to examine the binding modes and key protein-ligand interactions, and compared with that of the experimentally determined binding mode and interactions of the bound ligand, 5. The key interactions are mainly of hydrogen bonding interactions with Asp54, Ile14 and Leu164. The compounds that form at least one hydrogen bond interaction with the active site amino acid residues (particularly Asp54), and superimposing onto 1,3,5-triazine ring of 5 were considered as docking ligands whereas those compounds which failed to form hydrogen bond with the above residues, and whose 2,4-diaminopyrimidine ring did not superimpose onto 1,3,5-triazine ring of 5 were considered as non-docking ligands. In such compounds, all the docking conformations were found to be inconsistent with the X-ray conformation of 5. As it can be observed from their chemical structures (figs. 2 and 3), all the title compounds have 2,4-diamino functional groups which are expected to form key hydrogen bond interactions with Asp54 and other backbone amino acids residues (Ile14 and Leu164)[20,24–26,37,40]. Close observation of the poses of the docking ligands indicated that almost all of them have docking orientations similar to that of the bound ligand (5). Only the top scoring poses of these compounds were used for analyses. The Glide and FlexX scores of majority the compounds are higher than that of 5 whereas the GOLD fitness scores of almost all the compounds are either comparative or lower than the corresponding values of 5 (Table 1). Unless and otherwise mentioned specifically, all the discussions in this paper will be based on the Glide docking results obtained from the active site of quadruple mutant *Pf*DHFR enzyme.

**Fig. 4:**
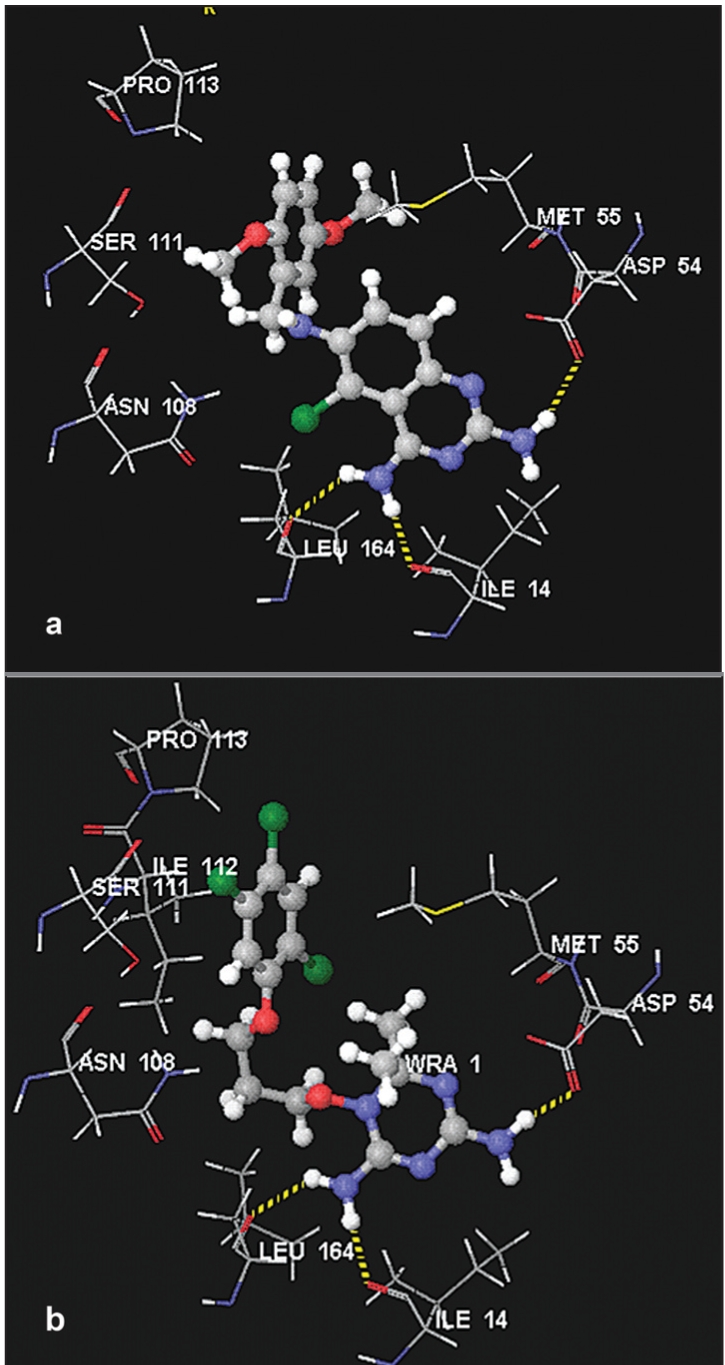
Stereoviews of the binding poses of 7 and 5 (a) Binding poses of compound 7 and (b) represents binding poses of compound 5. For the sake of clarity, only the important amino acid residues are given.

The docking pose analyses indicated that 2,4-diaminoquinazoline derivatives (7-13) have similar binding modes and comparative binding scores. Their Glide score values (range from -11.43 to - 11.97 kcal/mol) and FlexX scores (range from -22.08 to -25.56 kcal/mol) are much better than the corresponding scores of the bound ligand, 5, whereas their GOLD fitness scores are slightly lower than that of 5 (Table 1). A more negative value for Glide score and FlexX scores indicate better fit in the active site whereas for the GOLD fitness score a more positive value indicates a better fit in the binding site. Compounds 12 and 13 did not dock into the active site of the mutant *Pf*DHFR enzyme (Table 1). Unlike compounds 7-11, which have side chain on 6-position of the ring, 12 and 13 bear side chain at 5-position replacing Cl atom. The change in the position of the side chain from 6- to 5-position could be a reason why these two compounds are unable to dock into the active site. The trends in the binding modes and scores in the active site of wild type *Pf*DHFR enzyme are also similar to the corresponding observations for the quadruple mutant *Pf*DHFR enzyme (data not given). To illustrate the binding modes of this class of compounds in the active site of quadruple mutant *Pf*DHFR enzyme, the docking pose of most active compound (7) is briefly discussed as follows. The docking pose obtained from Glide software is used in this analysis. Fig. 4a shows the binding mode of 7 in the active site of quadruple mutant *Pf*DHFR whereas the corresponding binding mode of 5 is shown in fig. 4b. As shown in fig. 4a, 2-amino group of 7 forms an H-bond interaction with one of the carbonyl oxygen atoms of Asp54 whereas its 4-amino group interacts with Leu164 and Ile14 via H-bond interactions. The hydrogen bond lengths were 2.301, 2.303 and 1.949 Å, respectively. The bond length values of the corresponding H-bond interactions of 5 with Asp54, Leu164 and Ile14 are 2.000, 2.048 and 1.600 Å, respectively. The aromatic tail of the flexible side chain of 7 was observed to overlap partly on (trichlorophenoxy) propyloxy ring of 5. Thus, this aromatic moiety and its methoxy substituents showed hydrophobic interactions with amino acid residues such as Ser111, Pro113, Lys46 and Met55. These residues are also known to interact with trichloro phenyl ring of 5 via hydrophobic interactions[24,37]. Ring B of 7 is inserted between Phe58 and the cofactor (NADP), and expected to interact via aromatic-aromatic interactions which results in further stabilization of the inhibitor-enzyme complex. The Cl substituent was oriented into the vacant space between Leu164 and Asn108. It is 2.805 Å away from H-atom of amino group of Asn108, and is also expected to form additional weak H-bond interaction. Close examination of the ligand-protein complex also showed that there is no unfavorable steric interaction with Asn108 which is known to be responsible for resistance of the parasite towards 2 and 3[24,37].

**Fig. 5:**
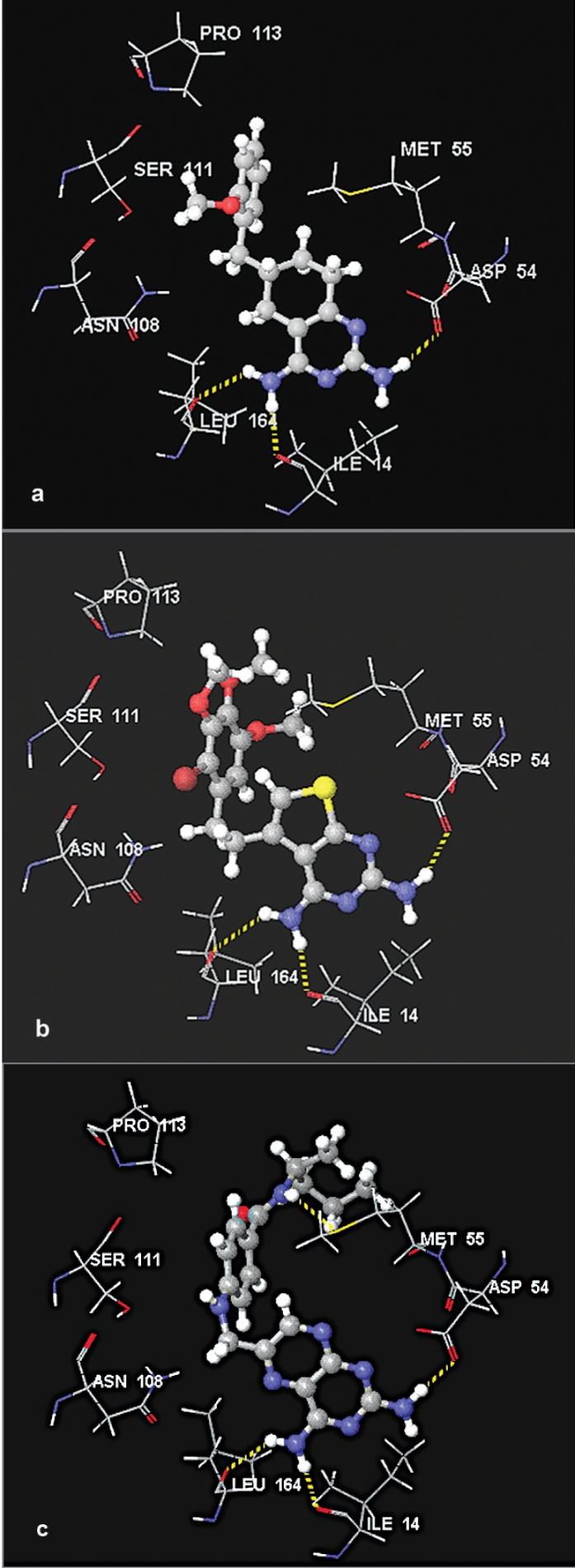
Stereoviews of the binding poses of 14, 19 and 21 (a) Binding poses of compound 14 (b) represents binding poses of compound 19 and (c) binding poses of compound 21. For the sake of clarity, only the important amino acid residues are given.

Visual inspection of docking poses of 2,4-diaminotetrahydroquinazolines (14-18) indicated that they bind into the active site of quadruple mutant *Pf*DHFR enzyme with similar binding modes. Their scores were also comparable to each other (Table 1). On the other hand, 18 was found to dock into the active site only during the FlexX calculation but failed to dock during the Glide and GOLD docking calculations (Table 1). To illustrate the binding modes of this series of compounds, 14 was selected for detail analysis. The binding mode of 14 is shown in fig. 5a. As illustrated in fig. 5a, compound 14 interacts with key amino acid residues in the active site via H-bonding interactions. Its 2-amino group forms an H-bond interaction with one of the carbonyl oxygen atoms of Asp54 whereas its 4-amino group interacts with Leu164 and Ile14 via H-bond interactions. The hydrogen bond length values are 1.974, 2.442 and 1.998 Å, respectively. These values are comparable to that of compound 7. This suggests that changing the aromatic ring B to non-aromatic ring, and also changing the linker unit to one-carbon atom has no significant effect on the docking modes and scores of this group of compounds (Table 1). The benzyl side chain (and its substituents) adopt orientations similar to trichlorophenyl group of 5 in such a way to avoid unfavorable interaction (especially with Asn108). This side chain also exhibits favorable hydrophobic interactions with residues such as Ser111, Pro113, Val45, Met55 and Phe116 near the opening of the active site similar to that of trichlorophenyl group of 5. π-stacking interaction between ring A of 14 and Phe58 was also maintained.

The 2,4-diaminothieno[2,3-d]pyrimidines (19-20) were also among the compounds used in the study. The visual inspection of the results indicated that these compounds showed similar docking modes with that of 5. They also showed all the necessary interactions exhibited by those compounds discussed above. Fig. 5b shows the binding mode of 19 in the active site of quadruple mutant *Pf*DHFR enzyme. Similar to that of compounds in the above series, the 2-amino group of 19 interacts with Asp54 whereas its 4-amino group interacts with backbone amino acid residues (Leu164 and Ile14) via H-bonding interactions. The corresponding H-bond length values are 2.153, 2.458 and 1.974 Å, respectively. The aromatic tail of 19 showed favorable hydrophobic interactions with amino acid residues near the opening of the active site, and also there is no potential steric clash with Asn108.

Analyses of the binding poses of 2,4-diaminopteridine derivatives (21-34) indicated that majority of the compounds have similar binding modes with each other and with that of 5 (Table 1). Compounds 26-29 did not dock into the active site during the GOLD docking calculation whereas compounds 32-34 did not dock at all into this active site as demonstrated by the results obtained from the three docking programs (Table 1). On the other hand, though they form H-bonding interactions with key amino acid residues, compounds 30-31 showed the lowest binding scores (Table 1). This indicated that long hydrophobic side chains play significant roles in enhancing binding interactions. This observation is consistent with the previous reports[40]. To illustrate the binding modes of this series of compounds, compound 21 was used as an example. Fig. 5c shows the binding mode of 21 in the active site of the quadruple mutant *Pf*DHFR enzyme. As it has been observed in the case of the other docking ligands discussed above, compound 21 also forms H-bonding interactions with the key amino acid residues. Its 2-amino group forms an H-bond interaction with one of the carbonyl oxygen atoms of Asp54 whereas the 4-amino group interacts with Leu164 and Ile14 via H-bond interactions. The observed hydrogen bond length values are 2.104, 2.104 and 1.745 Å, respectively. Moreover, the NH group of the linker chain forms one additional H-bond interaction with S-atom of Met55 (2.741 Å). The amino acid residues such as Pro113 and M55 were also observed to interact with the side chain via hydrophobic interactions. The orientation of the aromatic tail of the side chain is slightly tilted as compared to the tricholoro phenyl tail of 5. Therefore, no interaction was observed with Ser111. Moreover, no steric clash was observed with Asn108.

Structure-activity relationship (SAR) studies are commonly used in drug design and discovery of inhibitors of DHFR enzymes as well as understanding inhibitor-enzyme interactions[27–30,39,50–52]. In this paper, we present a brief discussion on the SARs of the title compounds. 2,4-diaminoquinazolines (7-13) and 2,4-diaminotetrahydroquinazolines (14-18) are among the group of compounds showing relatively high activities with pIC_50_ values ranging from 6.77 to 8.05 nM. Though these compounds are less active than 5 (pIC_50_, 8.57 nM), they are by far more potent than 3 (pIC_50_ ~ 5.38).

The trends in the activities of 2,4-diaminoquinazoline derivatives (7-13) suggested that the substitution patterns in the aromatic tails and the nature of the linker moieties play a significant role for the observed activity differences. For instance, compound 7 is more potent inhibitor than compound 8. The structural difference between these compounds is that in 7 the aromatic side chain bears two OMe groups at 2’- and 5’-positions whereas in the case of 8, there are three OMe groups at 3’-, 4’- and 5’-positions. This difference is believed to be responsible for the four-fold drop in the inhibitory activity of 8 as compared to 7[41]. When compared to each other, the structural difference between 8 and 9 is methylation of bridging N atom in 9. This structural difference does not result in a significant loss of activity suggesting a greater tolerance for substitution on the bridge nitrogen than at the *ortho*-position of the benzyl group. Elongation of the linker unit by one carbon (8 vs. 11) resulted in nearly 40-fold loss in the activity of 11 as compared to 8. For compounds 12 and 13, changing the substitution pattern on ring B from 5- to 6-positions could be responsible for the loss of potencies of these compounds as compared to 7, 8 and 9. It is important to note that the compounds have very similar docking scores (Table 1). Our attempt to correlate the observed docking scores with the reported biological activity (pIC_50_) values was not successful. The activities and docking scores of 2,4-diaminoquinazoline derivatives (14-18) are also similar to those of 2,4-diaminoquinazolines. They all have saturated ring B in their chemical structures (figs. 2 and 3). Compound 14 is the most active compound from this group followed by 15 and 16 with pIC_50_ values of 7.54, 7.44 and 7.30 nM, respectively (Table 1). Close observation of the chemical structures of these compounds indicated that substitution pattern and nature of the substituents on 6-benzyl side chain play an important role in determining activity. The 3’-OMe substituent enhances the inhibitory activity of 14 whereas moving OMe group from 3’- to 2’-position could cause a loss in activity (14 vs. 15). Replacing the 2’-OMe group by 2’-Me group (compound 16) results in two-fold loss of activity as compared to compound 14. The bulky substituents (OCF_3_) at 4’-position also results in 5-fold decrease in activity (17 vs. 14). Similar to that of 2,4-diaminoquinazolines, the docking scores are comparable (Table 1). Therefore, no attempt was made to correlate docking scores with biological activities.

On the other hand, the 2,4-diaminothieno [2,3-d] pyrimidines (19-20) and 2,4-diaminopteridine derivatives (21-34) are generally weak inhibitors with pIC_50_ values ranging from about 5.00 to 6.20 nM (Table 1).[41] Thus, their SARs are not discussed in detail in this paper. But based on their chemical structures, it is possible to make the following suggestions about their SARs. The suggestions are (i) amide substitution on the hydrophobic aromatic tail could be responsible for low inhibitory activities (e.g., 21-29) (ii) aromatic tails which could occupy hydrophobic portion of the binding region are required to enhance *Pf*DHFR inhibitory activities. The relatively low inhibitory activities of 30 and 31 support this argument. Though these compounds form key H-bond interactions with Asp54, Ile14 and Leu164, they show low inhibitory activities (Table 1). This could be attributed to lack of aromatic tails that interact via hydrophobic interactions with the enzyme near the opening of the active site. Previous reports suggest that antifolates with aromatic hydrophobic side chains exhibit enhanced inhibitory activities[40] and (iii) compounds with two bulky aromatic side chains are also weak inhibitors (e.g., 32-34). The presence of two bulky sides could be a reason that prevents the compounds from entering into the active site. This is consistent with the docking calculation results (Table 1).

In conclusion, the docking results indicated that most of the compounds adopt binding modes similar to that of the experimental bound ligand, 5, with better docking score values. Moreover, because of their flexible natures, the hydrophobic aromatic side chains were oriented in such a way that could avoid steric clash with Asn108 which is known to cause resistance toward the common antifolates. The molecular docking results and the SAR analyses obtained from this study would help medicinal chemists in designing antimalarial agents by employing target guided drug design approach. As a strategy, the following three points could be used to design ligands active against *Pf*DHFR enzyme. These are (i) polar head group that can form H-bond interactions with Asp54, Ile14 and Le164. This group could be aromatic or non-aromatic (ii) hydrophobic aromatic tails bearing substituent groups such as Cl and OMe. These tails occupy the hydrophobic pocket of the active site to enhance inhibitory activities and (iii) a linker unit between the polar head group and hydrophobic tail to provide flexibility to the ligand in order to avoid unfavorable steric clash with Asn108.
